# P-757. Does Dalbavancin Utilization in the ED Reduce Hospital Admission?

**DOI:** 10.1093/ofid/ofaf695.968

**Published:** 2026-01-11

**Authors:** Nadim Sidik, Jennifer Bui, Fermin Barrueto, Lisa Thomas, Russell D’Anton, Leonardo Girio-Herrera, Barbara Donithan

**Affiliations:** UM Upper Chesapeake Medical Center, Bel Air, MD; UM Upper Chesapeake Medical Center, Bel Air, MD; UM Upper Chesapeake Medical Center, Bel Air, MD; UM, Bel Air, Maryland; UM, Bel Air, Maryland; UM Upper Chesapeake Medical Center, Bel Air, MD; UM Upper Chesapeake Medical Center, Bel Air, MD

## Abstract

**Background:**

Dalbavancin is a parenteral long-acting lipoglycopeptide antibiotic that is FDA approved for acute bacterial skin and soft tissue infections (ABSSSI). It is effective against gram positive organisms including MSSA/MRSA, Streptococcus, and vancomycin susceptible Enterococcus. Its long half-life provides effective 7-day therapy with a single IV infusion. This unique quality is beneficial for patients whose cellulitis is severe enough to typically warrant initial IV therapy, and those who are unlikely to adhere to medications prescribed due to homelessness, lack of adequate insurance coverage/access to medications and people who inject drugs.

**Figure:**
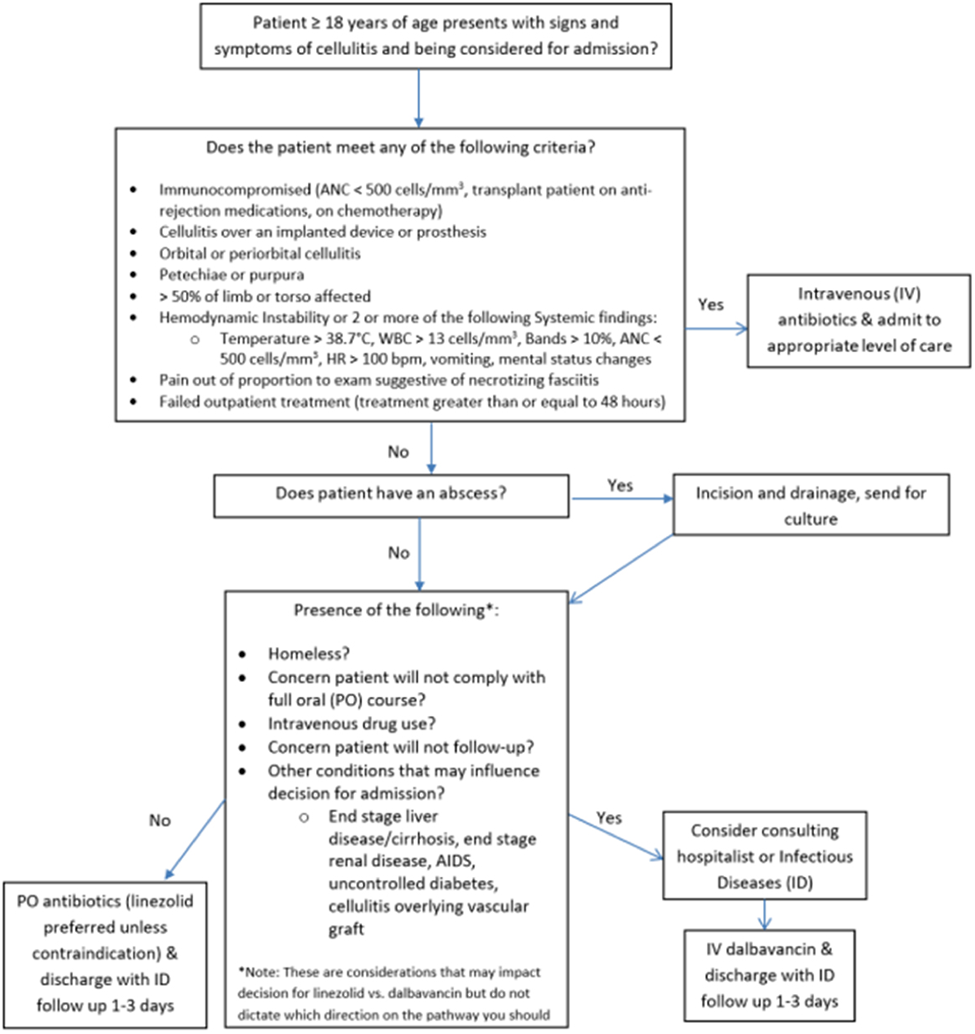
University of Maryland Medical System Dalbavancin Use Guideline Guideline used to establish appropriateness of dalbavancin usage.

**Table:**
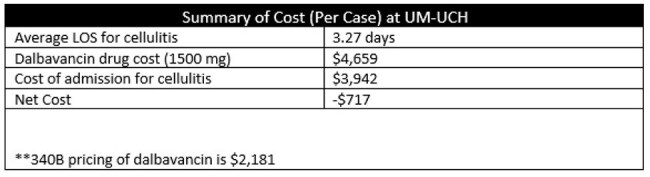
Cost Analysis

**Methods:**

We conducted a 6-month pilot from April 2024 to October 2024 including the ED at the University of Maryland (UM)– Upper Chesapeake Health, a community-based hospital with 316 licensed beds along with our affiliated, free-standing ED, Aberdeen Medical Center. Adult patients with a diagnosis of cellulitis without sepsis were evaluated by an ED provider in consultation with an ID provider when available, using specified criteria to determine eligibility for dalbavancin infusion. If a patient met criteria, a 1-time 1,500 mg dose was administered with subsequent discharge from the ED. Data were retrospectively collected and evaluated at the end of the pilot.

Overall Analysis

**Figure:**
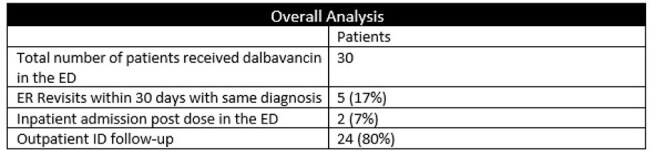

**Results:**

A total of 30 patients met criteria and received dalbavancin in our ED during the pilot. Our dalbavancin pathway avoided 25 admissions. We calculated a savings of 81 patient days during the 6-month pilot. Although we found a net cost increase of $717 per patient (vs inpatient admission), there was an overall savings as reduced cellulitis admissions created bed availability for other patient admissions and helped reduce the number of ED boarders.

**Conclusion:**

A single-dose dalbavancin infusion given to eligible patients in the ED avoids admissions to the hospital for patients with ABSSSI with suspected or documented gram-positive organisms. Cost savings from avoiding hospital stay did not offset the cost of dalbavancin in our hospital. However, hospitals with 340B pricing are expected to have cost saving in addition to reducing hospital admissions. Dalbavancin should be judiciously used, and alternative oral antibiotic options should be pursued when appropriate.

**Disclosures:**

All Authors: No reported disclosures

